# Enzyme Activity in the Crowded Milieu

**DOI:** 10.1371/journal.pone.0039418

**Published:** 2012-06-26

**Authors:** Tobias Vöpel, George I. Makhatadze

**Affiliations:** Department of Biology and Center for Biotechnology and Interdisciplinary Studies, Rensselaer Polytechnic Institute, Troy, New York, United States of America; Nagoya University, Japan

## Abstract

The cytosol of a cell is a concentrated milieu of a variety of different molecules, including small molecules (salts and metabolites) and macromolecules such as nucleic acids, polysaccharides, proteins and large macromolecular complexes. Macromolecular crowding in the cytosolic environment is proposed to influence various properties of proteins, including substrate binding affinity and enzymatic activity. Here we chose to use the synthetic crowding agent Ficoll, which is commonly used to mimic cytosolic crowding conditions to study the crowding effect on the catalytic properties of glycolytic enzymes, namely phosphoglycerate kinase, glyceraldehyde 3-phosphate dehydrogenase, and acylphosphatase. We determined the kinetic parameters of these enzymes in the absence and in the presence of the crowding agent. We found that the Michaelis constant, K_m_, and the catalytic turnover number, k_cat_, of these enzymes are not perturbed by the presence of the crowding agent Ficoll. Our results support earlier findings which suggested that the Michaelis constant of certain enzymes evolved in consonance with the substrate concentration in the cell to allow effective enzyme function in bidirectional pathways. This conclusion is further supported by the analysis of nine other enzymes for which the K_m_ values in the presence and absence of crowding agents have been measured.

## Introduction

The interior of cells, namely the cytosol, is not only filled with water and salts but also with a variety of different soluble metabolites and macromolecules (proteins, nucleic acids, oligosaccharides). The concentration of macromolecules can vary between 200–400 g/l depending on the organism (eukaryotes vs. prokaryotes) [1], [2]. Recently the intracellular metabolite pool of *E. coli* was assessed to have an approximate concentration of 300 mM [3].

All of these solute macromolecules have an influence on each other and might affect the mobility, stability, association property, and activity of proteins [4]. The effect of macromolecules on each other, also known as macromolecular crowding or the excluded volume effect, has been studied extensively over the last decade [5], [6]. Many different aspects of crowding have been discussed including the thermal stabilization of flavodoxin by synthetic crowders such as Ficoll [7], or the thermal stabilization of lens crystallin at high concentrations of protein crowders [8]. Interestingly, a recent study by Miklos *et al.* shows that the synthetic crowder PVP can stabilize the protein Cl2 in contrast to protein crowders such as bovine serum albumin or lysozyme which destabilize this protein [9]. In terms of protein association, different effects of crowding have been reported. Crowders were shown to enhance polymerization, self-association, and hetero-oligomerization [10], [11], [12], [13]. On the other hand, crowders have little effect on the association of heterodimers in other model systems [14]. The studies of the effects of crowding on enzyme activity have also produced opposing results, as most studies were focused on the effects of crowding agents on the specific activity [15], [16].

In this study we examined the effects of a crowding agent on the kinetic parameters of three different enzymes (yeast phosphoglycerate kinase - PGK, rabbit muscle glyceraldehyde 3-phosphate dehydrogenase – GAPDH, and human acylphosphatase 1 - ACP) in the terms of changes in the Michaelis constant, K_m_. This was inspired by Bennett *et al.* [3] who investigated the influence of intracellular metabolite concentrations on the active-site occupancy of enzymes. In their work, the authors compared the K_m_ values of enzymes (as compiled in BRENDA information system [17]) with the measured intracellular substrate concentrations. They find that the K_m_ values are directly correlated (with a slope of one) to the corresponding substrate concentrations for several major classes of enzymes, including enzymes involved in carbon metabolism [3]. It is noted, however, that the reported K_m_ values in BRENDA are based on studies performed in dilute aqueous solution, i.e. in the absence of crowding agents. Nevertheless, this study suggests that the Michaelis constant is directly related to the available substrate concentration in the metabolite pool and that thermodynamic constraints dictate the effective cellular enzymatic activity. The question is whether the crowded cellular environment significantly affects the K_m_ values of enzymes, and thus the correlation between the K_m_ and cellular substrate concentrations, as observed by Bennett *et al.* [3], is circumstantial.

We find that the addition of 200 g/l of Ficoll, a neutral polymer that is often used to mimic crowded cellular environment, has a very small effect on K_m_ of the three studied enzymes, PGK, GAPDH, and ACP. Analysis of the published data for several other enzymes further supports this finding. Overall, our results support the hypotheses put forward by Bennett *et al.* [3] that to ensure a rapid response to the changes in the metabolic flux, enzymes have Michaelis constants close to the cellular substrate concentrations.

## Materials and Methods

### Reagents

All reagents were obtained in the highest purity from Sigma-Aldrich Co. LLC. (USA). Ficoll PM 70 was obtained from GE Healthcare Bio-Sciences Corp. (USA).

### Protein Preparations

Yeast PGK wt with an N-terminal 6×His-Tag followed by a TEV restriction site was expressed from a pGia [18] vector in *Escherichia Coli* strain BL21 (DE3) pLys. The cells were grown in TB-media at 37°C until the OD_600_ reached 0.8. The temperature was then decreased to 25°C and protein expression was induced by the addition of 250 µM Isopropyl β-D-1-thiogalactopyranoside (IPTG) overnight. The cells were harvested and disrupted using a French Laboratory Press (Thermo Fisher). Cell debris was pelleted at 8,900 rpm and 4°C. The supernatant was applied to a column packed with Ni-NTA resin (Novagen). The column was washed with buffer containing 50 mM potassium phosphate (KPi), 300 mM sodium chloride (NaCl), 10 mM Imidazole pH 7.4 until the OD_280_ reached a minimum baseline. The protein was then eluted from the column using a buffer containing 300 mM Imidazole. The protein solution was immediately dialyzed against a buffer containing 50 mM KPi, 300 mM NaCl, 0.5 mM ethylenediaminetetraacetic acid (EDTA) and 1 mM dithiothreitol (DTT) at 4°C for 2 hours. To cleave off the N-terminal 6×His-Tag, TEV protease [19] was added directly to the dialysis bag. The reaction mixture was left on dialysis overnight. The uncleaved protein and the TEV protease were removed by reapplying the reaction mixture to the Ni-NTA column. The flow through containing PGK was concentrated using an Amicon Ultra-15 Centrifugal Filter Unit (EMD Millipore, MA). Protein purity was verified by SDS-PAGE. The concentration was determined by the protein absorbance at 280 nm (ε_280_ (yPGK) = 21,360 M^−1^ cm^−1^) in buffer containing 20 mM KPi, 6 M guanidinium hydrochloride, pH 6.5 according to the method described by Gill and von Hippel [20]. Expression and purification of acylphosphatase was performed as described earlier [21]. Rabbit muscle glyceraldehyde 3-phosphate dehydrogenase (GAPDH, EC: 1.2.1.12) was purchased from Sigma-Aldrich Co. LLC. (USA).

### Phosphoglycerate Kinase Activity

**Figure 1:**
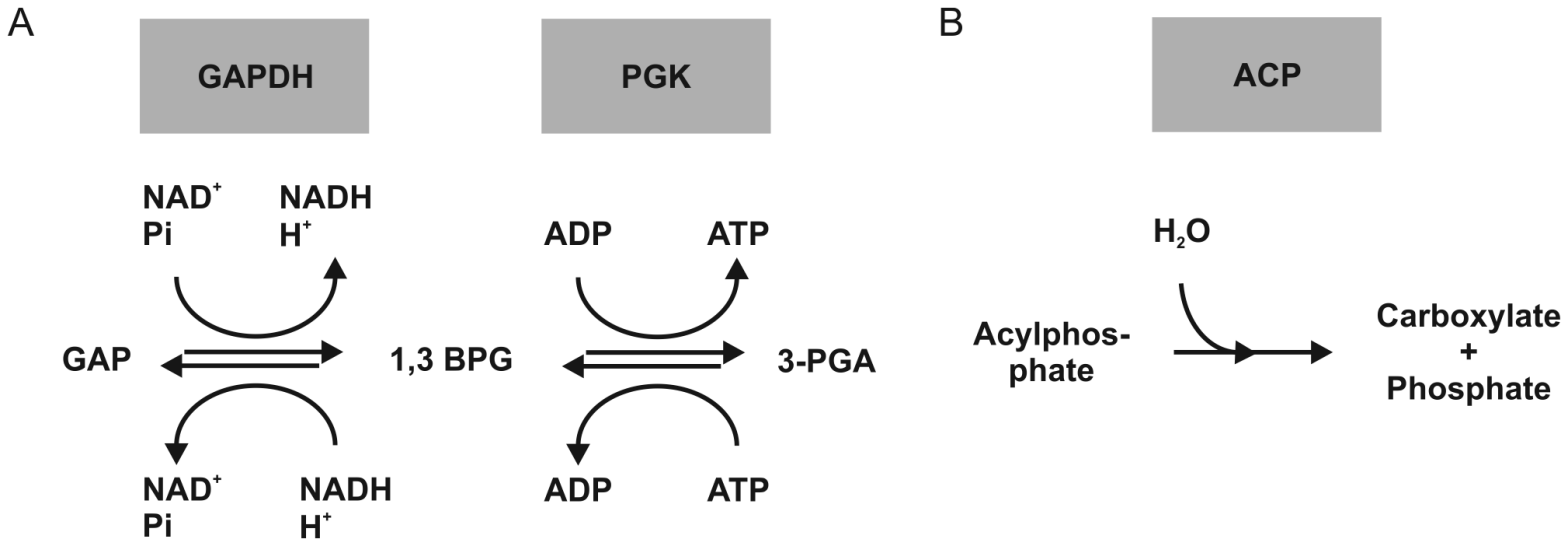
Reaction schemes for the tested enzymes. (**A**) Reactions in the glucose metabolism catalyzed by GAPDH and PGK. In the forward reaction for GAPDH, conversion of substrate GAP is monitored directly by changes in NADH concentration. In the forward reaction for PGK, conversion of substrate ADP is monitored in a linked assay with forward reaction of GAPDH as a source for 1,3 BPG. In the reverse reaction for PGK, conversion of 3-PGA is monitored by the changes in NADH concentration in a linked assay with 1,3 BPG as a substrate for GAPDH [22]. (**B**) Reaction catalyzed by ACP. Hydrolysis of acylphosphate leads to the formation of a carboxylate and an inorganic phosphate.

The enzymatic activity of yeast PGK was monitored using a coupled reaction with GAPDH [22]. The reaction rates for different concentrations of the substrates (3-phosphoglycerate (3-PGA) or adenosine diphosphate (ADP)) were determined from the changes in the concentration of reduced nicotinamide adenine dinucleotide (NADH). The concentration of NADH was monitored spectrophotometrically at 340 nm (ε_340_ (NADH) = 6,220 M^−1^ cm^−1^). The reaction rates were obtained by analyzing the initial slope of changes in absorbance during the first 5 seconds. The forward reaction (Figure 1A) was carried out in 50 mM Tris, 50 mM KPi, 3 mM magnesium chloride (MgCl_2_), 1 mM EDTA, 1.5 mM DTT at pH 7.4 with and without 200 g/l Ficoll PM70. The concentrations of glyceraldehyde-3-phosphate (GAP) and nicotinamide adenine dinucleotide (NAD^+^) were 830 µM and 415 µM, respectively and the ADP concentration was varied between 12.5–3,000 µM. For the reverse reaction (Figure 1A), 50 mM Tris, 3 mM MgCl_2_, 1 mM EDTA, and 1.5 mM DTT at pH 7.4 with or without 200 g/l Ficoll PM70 was used as a buffer. The concentrations of ATP and NADH were 2,500 µM and 100 µM, respectively, while the concentration of 3-PGA was varied between 250–30,000 µM. In all reactions, the concentration of PGK was 5 nM and the concentration of GAPDH was 1 µM. The observed rates were normalized to the concentration of PGK.

### Glyceraldehyde 3-phosphate Dehydrogenase Activity

The enzymatic activity of GAPDH was monitored by the increase in NADH concentration (ε_340_ (NADH) = 6,220 M^−1^ cm^−1^). The initial slope of the reaction covering the first 5 seconds was used for analysis. The forward reaction was carried out in 50 mM Tris, 50 mM KPi, 3 mM magnesium chloride (MgCl_2_), 1 mM EDTA, 1.5 mM DTT at pH 7.4 without or with the addition of 200 g/l Ficoll PM70. The concentration of NAD^+^ was 500 µM, while the concentration of glyceraldehyde-3-phosphate (GAP) was varied between 125–10,000 µM. In all reactions, the concentration of GAPDH was 20 nM. The observed rates were normalized to the concentration of GAPDH.

### Acylphosphatase 1 Activity

The enzymatic activity of ACP was monitored by a decrease in absorbance upon hydrolysis of benzoyl phosphate (BP) using an extinction coefficient of ε_283_ (BP) = 960 M^−1^ cm^−1^ [23]. The initial slope of the reaction covering the first 5 seconds was used for analysis. The reaction was carried out in 100 mM sodium acetate pH 5.5 with or without the addition of 200 g/l Ficoll PM70. The concentration of the substrate benzoyl phosphate was varied between 25–1,000 µM. In all reactions, the ACP concentration was 10 nM. The observed rates were normalized to the concentration of ACP.

### Michaelis-Menten Kinetics

All activity measurements were performed in triplicate using an SX.18MV-R stopped-flow apparatus (Applied Photophysics Ltd, UK) at 25°C. The initial reaction rates at different substrate concentrations were analyzed by nonlinear regression fit to Michaelis-Menten equation (Eq. 1) using Origin:
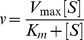
(1)Where ν is the reaction rate, V_max_ is the maximum reaction rate, K_m_ is the Michaelis constant and [S] is the substrate concentration.

## Results and Discussion

### Phosphoglycerate Kinase Activity for ADP as a Substrate

The rate of the yeast PGK activity in the forward reaction (See Figure 1A) versus the ADP substrate concentration is shown in Figure S1A. Nonlinear regression analysis according to the Michaelis-Menten equation gives the following kinetic parameters of the reaction: K_m_ = 340±40 µM and k_cat_ = 860±40 s^−1^. These results are in agreement with earlier studies on yeast PGK which reported similar kinetic parameters under comparable conditions with a K_m_ value of 180 and a k_cat_ of 963 s^−1^ [24] or a K_m_ = 500 µM and k_cat_ of 613 s^−1^ [25]. The kinetic parameters for yeast PGK are also comparable to the kinetic parameters of PGK from different organisms (see supplementary Table S1 for comparison).

We also performed the activity measurements in the presence of 200 g/l Ficoll to elucidate the effects of the crowding agent on the kinetic parameters. Figure S1B shows the Michaelis-Menten plot for the forward reaction in the presence of Ficoll. The K_m_ for ADP as the substrate was 430±40 s^−1^ µM, while the k_cat_ was 920±30 s^−1^. These kinetic parameters obtained in the presence of the crowding agent Ficoll are similar to the parameters obtained in the absence of Ficoll, suggesting that the crowding agent does not have a significant effect on the activity of PGK (also see Table 1).

**Table 1:** Kinetic parameters of PGK, GAPDH and ACP in the absence or presence of the crowding agent Ficoll.

Substrate	Without Crowder	With Crowder
PGK		
**ADP**		
K_m_ (µM)	340±40	430±40
k_cat_ (s^−1^)	860±40	920±30
**3-PGA**		
K_m_ (µM)	3300±500	2000±300
k_cat_ (s^−1^)	490±30	490±20
GAPDH		
**GAP**		
K_m_ (µM)	1000±160	1100±160
k_cat_ (s^−1^)	50±3	37±2
ACP		
**Benzoyl phosphate**		
K_m_ (µM)	100±18	80±17
k_cat_ (s^−1^)	1100±60	300±17

### Phosphoglycerate Kinase Activity for 3-PGA as a Substrate

In Figure S2, the rate of PGK in the reverse reaction (See Figure 1A) is plotted versus the 3-PGA substrate concentration. The substrate concentration was varied between 250–30,000 µM. The kinetic parameters from Michaelis- Menten analysis (Equation 1) were: K_m_ =  3,300±500 µM and k_cat_ =  490±30 s^−1^. In the presence of the crowding agent Ficoll we do not observe a change in k_cat_, 490±20 s^−1^ in comparison to the absence of the crowding agent (490±30 s^−1^). Similarly, there is an insignificant decrease in the K_m_ value (2,000±300 µM) between the crowded and uncrowded conditions. Thus, the activity of PGK for 3-PGA as a substrate is not influenced by the addition of the crowding agent Ficoll. This is consistent with the effect of the crowding on the activity of PGK for the other substrate, ADP (see Table 1). If we assume that Ficoll is a suitable mimic of the crowded environment in the cell, our results suggest that the activity of PGK in the cell should be similar to the activity observed in vitro.

**Table 2:** Crowding agent effects on the kinetic parameter, K_m_, of different enzymes.

Enzyme	Crowding Agent	Conc. (g/l)	Substrate	K_m_ change	Reference
ACP	Ficoll	200	Benzoyl phosphate	1.3	This work
GAPDH	Ficoll	200	GAP	0.9	This work
PGK	Ficoll	200	ADP	0.9	This work
	Ficoll	200	3-PGA	1.7	This work
AspP	PEG 6000	50	ADP-glucose	3.3	[31]
EntB	Ficoll	300	Isochorismate	1.6	[32]
EntC	Ficoll	300	Chorismate	2.2	[32]
HK	BSA	200	Glucose	1.3	[33]
Hyal	PEG 4000	50	Hyaluronic acid	1.4	[34]
LDH	Dextran 40,000	200	Lactate	1.8	[34]
		200	NAD^+^	1.6	[34]
		200	Pyruvate	1.9	[34]
LDH	Ficoll	300	Pyruvate	3.1	[32]
MCO	Ficoll/Dextran	200	o-Dianisidine HCL	0.1	[35]
MenF	Ficoll	300	Chorismate	2.5	[32]
Trypsin	Dextran 40,000	257	Benzoyl-arginine-p-nitroanilide	1.4	[34]

The K_m_ change is represented as the ratio of the K_m_ in the absence of the crowding agent divided by the K_m_ in the presence of the crowding agent. AspP – ADP-sugar pyrophosphatase; EntB – isochorismatase; EntC – isochorismate synthase; HK – hexokinase; Hyal – hyaluronate lyase; LDH – lactate dehydrogenase, MCO – multi-copper oxidase; MenF – monomeric isochorismate synthase.

### Glyceraldehyde 3-phosphate Dehydrogenase Activity

Figure S3 shows the activity measurements of GAPDH in the absence and presence of Ficoll to assess the influence of a crowded environment. The activity measurements were performed in the forward reaction (Figure S1A) by varying the concentration of the substrate GAP between 125–10,000 µM. The K_m_ value obtained from the Michaelis-Menten plot in the absence of Ficoll is 1000±160 µM and the k_cat_ value is 50±3 s^−1^. In the presence of Ficoll, the values of the kinetic parameters are K_m_ =  1,100±150 µM and k_cat_ =  37±2 s^−1^. The small decrease in k_cat_ in the presence of Ficoll is related to the changes in the monomer-tetramer equilibrium of GAPDH in the presence of the crowding agent. It has been shown that crowding increases the association constant of tetramer formation in GAPDH, which will have an indirect effect on the activity, as the tetramer has a 30 times lower activity than the monomer [26]. Importantly, the K_m_ value did not change in the presence of the crowding agent. This is in agreement with the observations we made for PGK, where the Michaelis constant also does not change in the presence of Ficoll.

### Acylphosphatase 1 Activity

The activity of ACP was directly assayed using the model substrate benzoyl phosphate [23]. The reaction scheme is depicted in Figure S1B. The substrate concentration was varied between 25–1,000 µM (Figure S4). The K_m_ and k_cat_ values were determined from a fit to the Michaelis-Menten equation (Equation 1). The K_m_ value is 100±18 µM, while the k_cat_ value is 1100±60 s^−1^ at 25°C, in agreement with previous measurements [18]. We also measured the activity of ACP in the presence of 200 g/l Ficoll PM70. The k_cat_ value in the presence of Ficoll is only three times lower (300±17 s^−1^) than in the absence of Ficoll. The K_m_ value in the presence of Ficoll is 80±17 µM, which is within experimental error of the K_m_ obtained in the absence of crowding agent. Since the kinetic parameters for benzoyl phosphate are similar in the absence and presence of crowding agent, we can assume that they will also be similar for the natural substrate acetyl phosphate. It is known that the K_m_ for acetyl phosphate is 30 times higher than for benzoyl phosphate, which puts an estimate for K_m_ for this substrate at 2,900 µM [27].

### Relevance to the *in vivo* Activity

The activities for three different enzymes were analyzed in the presence of the crowding agent Ficoll to mimic a cell like environment with a high concentration of macromolecules. This was done to evaluate potential differences in their enzymatic activity *in vitro* (in dilute aqueous solution) and *in vivo* (in the crowded environment). Our goal was to investigate if the kinetic parameters were altered in the presence of the crowding agent. We have shown that the presence of a crowding agent does not influence the kinetic parameters and in particular the K_m_ for PGK, GAPDH and ACP. The K_m_, in a first approximation, is the dissociation constant of the Michaelis complex and, thus, defines the fraction of the enzyme-substrate complex at a given substrate concentration. When the substrate concentration is equal to K_m_, only half of the enzyme is in the enzyme-substrate complex. For the enzymes involved in central carbon metabolism, it has been suggested that they have a K_m_ value which is not significantly different (within 10-fold) from their substrate concentration to ensure rapid enzyme response to changes in substrate concentration in either direction [3], [28], [29]. However, these observations were made using K_m_ values measured *in vitro*. Our measurements show that the activities of PGK, GAPDH and ACP in the absence or presence of the crowding agent Ficoll (which is used to mimic the crowded cell interior) are similar, thus, supporting this idea.

Indeed, the concentration of ADP, a substrate for PGK, is 560 µM in exponentially growing *E. coli* [3]. Our measured K_m_ values are less than 2-fold lower than the cellular concentrations of ADP (Table 1). The reported concentration of 3-PGA, another PGK substrate, in *E. Coli* is 1,500 µM [3]. The K_m_ value measured for PGK in the presence of Ficoll is similar to the cellular concentration of 3-PGA (Table 1). The concentration of GAP in *E. Coli* is 1,200 µM [30], and our results suggests that GAPDH also has a K_m_ value which is similar to the corresponding substrate concentration (Table 1). Finally, the concentration of the natural substrate for ACP, acetyl phosphate, in *E. Coli* is 1,100 µM [3]. The K_m_ of ACP for acetyl phosphate is 2,900 µM [27] and, as we discussed above, this value should not be affected by the crowded cellular environment. For all three studied enzymes, the cellular concentrations of the substrates and the K_m_ values are very similar, which allows the enzymes to provide an immediate response to the changes in the substrate concentration. PGK and GAPDH are of special interest because both enzymes are part of central carbon metabolism and, therefore, perform their activity in two directions as needed. Our observations underline the hypothesis by Bennett *et al.* which proposes that the substrate binding sites of the enzymes involved in bidirectional carbon metabolism cannot be fully saturated with their corresponding substrates in order to allow efficient catalysis in both directions [3]. Such a conclusion is not limited to the three enzymes studied here. Table 2 shows a compilation of the known effects of various crowding agents on the K_m_ values of nine other enzymes. Some of these enzymes are also involved in both catabolic and anabolic metabolism (e.g. lactate dehydrogenase and hexokinase) but others are only involved in catabolic processes (e.g. trypsin, hyaluronate lyase). Nevertheless, the K_m_ values for all of these enzymes are not significantly affected by the presence of crowding agents.

To summarize, we conclude that 1. crowding agents that mimic the cellular environment insignificantly affect the K_m_ of enzymes, and 2. cellular concentrations of many substrates are very similar to the K_m_ values of the corresponding enzymes in the presence of crowding agents. These two findings support the previous idea that many enzymes in the cell are always under conditions where they can efficiently respond to the changes in the concentration of the substrate and, thus, provide an efficient and simple initial regulation of the flux of metabolites.

## Supporting Information

Figure S1 **Michaelis-Menten plots for PGK in the forward reaction.** (**A**) Michaelis-Menten plot for PGK (5 nM) with varying concentrations of ADP in the absence of Ficoll. (**B**) Michaelis-Menten plot for PGK (5 nM) with varying concentrations of ADP in the presence of Ficoll. The black lines represent the fit to the Michaelis-Menten equation. The fit results are collated in Table 1.
(PDF)

Figure S2 **Michaelis-Menten plots for PGK in the reverse reaction.** (**A**) Michaelis-Menten plot for PGK (5 nM) with varying concentrations of 3-PGA in the absence of Ficoll. (**B**) Michaelis-Menten plot for PGK (5 nM) with varying concentrations of 3-PGA in the presence of Ficoll. The black lines represent the fit to the Michaelis-Menten equation. The fit results are collated in Table 1.
(PDF)

Figure S3 **Michaelis-Menten plots for GAPDH in the forward reaction.** (**A**) Michaelis-Menten plot for GAPDH (20 nM) with varying concentrations of GAP in the absence of Ficoll. (**B**) Michaelis-Menten plot for GAPDH (20 nM) with varying concentrations of GAP in the presence of Ficoll. The black lines represent the fit to the Michaelis-Menten equation. The fit results are collated in Table 1.
(PDF)

Figure S4 **Michaelis-Menten plots for ACP with varying concentrations of benzoyl phosphate.** (**A**) Michaelis-Menten plot for ACP (10 nM) with varying concentrations of benzoyl phosphate in the absence of Ficoll. (**B**) Michaelis-Menten plot for ACP (10 nM) with varying concentrations of benzoyl phosphate in the presence of Ficoll. The black lines represent the fit to the Michaelis-Menten equation. The fit results are collated in Table 1.
(PDF)

Table S1 **Summary of K_m_ values for PGK from different species.**
(PDF)
