# Supplementary material for: Enzyme Activity in the Crowded Milieu
Source: PLoS One. 2012 Jun 26;7(6):e39418. doi: 10.1371/journal.pone.0039418 (PMC3383682; doi:10.1371/journal.pone.0039418)
Supplement: Figure S2 — Michaelis-Menten plots for PGK in the reverse reaction. (A) Michaelis-Menten plot for PGK (5 nM) with varying concentrations of 3-PGA in the absence of Ficoll. (B) Michaelis-Menten plot for PGK (5 nM) with varying concentrations of 3-PGA in the presence of Ficoll. The black lines represent the fit to the Michaelis-Menten equation. The fit results are collated in Table 1. (PDF) [file pone.0039418.s002.pdf]

# Enzyme Activity in the Crowded Milieu

*Tobias Vöpel and George Makhatadze*

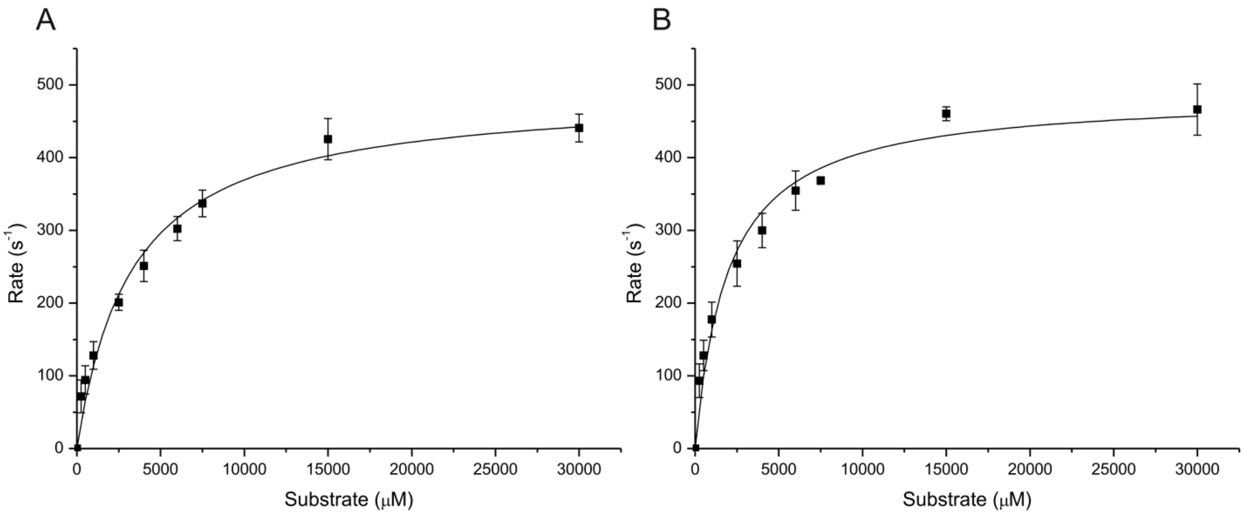


**Figure S2 Michaelis-Menten plots for PGK in the reverse reaction.**
**(A)** Michaelis-Menten plot for PGK (5 nM) with varying concentrations of 3-PGA in the absence of Ficoll. **(B)** Michaelis-Menten plot for PGK (5 nM) with varying concentrations of 3-PGA in the presence of Ficoll. The black lines represent the fit to the Michaelis-Menten equation. The fit results are collated in Table 1.
