# Supplementary material for: Enzyme Activity in the Crowded Milieu
Source: PLoS One. 2012 Jun 26;7(6):e39418. doi: 10.1371/journal.pone.0039418 (PMC3383682; doi:10.1371/journal.pone.0039418)
Supplement: Figure S3 — Michaelis-Menten plots for GAPDH in the forward reaction. (A) Michaelis-Menten plot for GAPDH (20 nM) with varying concentrations of GAP in the absence of Ficoll. (B) Michaelis-Menten plot for GAPDH (20 nM) with varying concentrations of GAP in the presence of Ficoll. The black lines represent the fit to the Michaelis-Menten equation. The fit results are collated in Table 1. (PDF) [file pone.0039418.s003.pdf]

Enzyme Activity in the Crowded Milieu

Tobias Vöpel and George Makhatadze

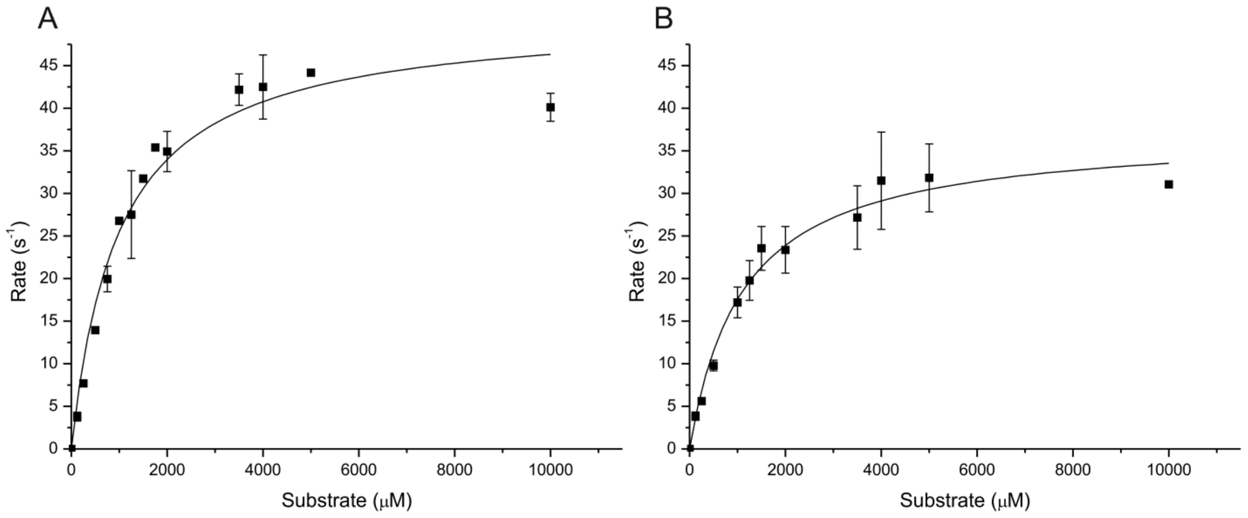

Figure S3 Michaelis-Menten plots for GAPDH in the forward reaction.
(A) Michaelis-Menten plot for GAPDH (20 nM) with varying concentrations of GAP in the absence of Ficoll. **(B)** Michaelis-Menten plot for GAPDH (20 nM) with varying concentrations of GAP in the presence of Ficoll. The black lines represent the fit to the Michaelis-Menten equation. The fit results are collated in Table 1.
