# Supplementary material for: Enzyme Activity in the Crowded Milieu
Source: PLoS One. 2012 Jun 26;7(6):e39418. doi: 10.1371/journal.pone.0039418 (PMC3383682; doi:10.1371/journal.pone.0039418)
Supplement: Table S1 — Summary of Km values for PGK from different species. (PDF) [file pone.0039418.s005.pdf]

Enzyme Activity in the Crowded Milieu

Tobias Vöpel and George Makhatadze

Table S1 Summary of K_m values for PGK from different species.

Source	K_m (μM)	Reference
S. cerevisiae		
3-PGA	770-1280	[1,2]
ADP	180-500	[1,3,4]
O. cuniculus		
3-PGA	1370	[5]
ADP	150	[5]
S. oleracea		
3-PGA	1100	[1]
ADP	270	[1]
H. Sapiens		
3-PGA	100-850	[5,6,7]
ADP	83-120	[5,6]

References

1. *Kuntz GW, Krietsch WK (1982) Phosphoglycerate kinase from spinach, blue-green algae, and yeast. Methods Enzymol 90 Pt E: 110-114.*
2. *McHarg J, Kelly SM, Price NC, Cooper A, Littlechild JA (1999) Site-directed mutagenesis of proline 204 in the 'hinge' region of yeast phosphoglycerate kinase. Eur J Biochem 259: 939-945.*
3. *Hurth C, Tassius C, Talbot JC, Maali A, Moskalenko C, et al. (2007) Enzymatic activity of immobilized yeast phosphoglycerate kinase. Biosens Bioelectron 22: 2449-2455.*
4. *Mas MT, Bailey JM, Resplandor ZE (1988) Site-directed mutagenesis of histidine-388 in the hinge region of yeast 3-phosphoglycerate kinase: effects on catalytic activity and activation by sulfate. Biochemistry 27: 1168-1172.*
5. *Kuntz GW, Krietsch WK (1982) Phosphoglycerate kinase from animal tissue. Methods Enzymol 90 Pt E: 103-110.*
6. *Szabo J, Varga A, Flachner B, Konarev PV, Svergun DI, et al. (2008) Communication between the nucleotide site and the main molecular hinge of 3-phosphoglycerate kinase. Biochemistry 47: 6735-6744.*
7. *Yoshida A (1975) Human phosphoglycerate kinase. Methods Enzymol 42: 144-148.*
